# Spatial multi-scaled chimera states of cerebral cortex network and its inherent structure-dynamics relationship in human brain

**DOI:** 10.1093/nsr/nwaa125

**Published:** 2020-06-05

**Authors:** Siyu Huo, Changhai Tian, Muhua Zheng, Shuguang Guan, Changsong Zhou, Zonghua Liu

**Affiliations:** State Key Laboratory of Precision Spectroscopy and Department of Physics, East China Normal University, Shanghai 200062, China; State Key Laboratory of Precision Spectroscopy and Department of Physics, East China Normal University, Shanghai 200062, China; School of Data Science, Tongren University, Tongren 554300, China; State Key Laboratory of Precision Spectroscopy and Department of Physics, East China Normal University, Shanghai 200062, China; State Key Laboratory of Precision Spectroscopy and Department of Physics, East China Normal University, Shanghai 200062, China; Department of Physics, Centre for Nonlinear Studies and Beijing-Hong Kong-Singapore Joint Centre for Nonlinear and Complex Systems (Hong Kong), Institute of Computational and Theoretical Studies, Hong Kong Baptist University, China; Department of Physics, Zhejiang University, Hangzhou 310058, China; State Key Laboratory of Precision Spectroscopy and Department of Physics, East China Normal University, Shanghai 200062, China

**Keywords:** synchronization, chimera state, cerebral cortex network, neural mass model

## Abstract

Human cerebral cortex displays various dynamics patterns under different states, however the mechanism how such diverse patterns can be supported by the underlying brain network is still not well understood. Human brain has a unique network structure with different regions of interesting to perform cognitive tasks. Using coupled neural mass oscillators on human cortical network and paying attention to both global and local regions, we observe a new feature of chimera states with multiple spatial scales and a positive correlation between the synchronization preference of local region and the degree of symmetry of the connectivity of the region in the network. Further, we use the concept of effective symmetry in the network to build structural and dynamical hierarchical trees and find close matching between them. These results help to explain the multiple brain rhythms observed in experiments and suggest a generic principle for complex brain network as a structure substrate to support diverse functional patterns.

## Teaser

Using the neural mass model on human cortical network, we reveal a new chimera state with multiple spatial scales and provide a way to explain the multiple brain rhythms observed in experiments.

## INTRODUCTION

Cerebral cortex in the brain displays various dynamical patterns under different states in normal functioning and neuropsychiatric disorders. It is now well known from EEG data that phase synchronization occurs among distributed functional regions involved in different cognitive processes [1,2]. For example, synchronization in theta band is related to short-term (episodic) memory, whereas a task-specific desynchronization in the upper alpha band is related to long-term (semantic) memory [3]. Thus, local synchronization between specific cortical regions plays key roles in normal cognitive dynamics [4]. During sleeping or anaesthesia, the brain activity patterns shift to large-scale, apparently cortex-wide synchronous up-down states [5]. Such synchronous up-down states can also happen locally, leading to local sleep [6]. Recently, a new phenomenon in human sleep has been revealed, called *the first-night effect* (FNE) [7], which represents troubled sleep in a novel environment. In the FNE, one hemisphere is more vigilant than the other in unfamiliar surroundings during sleep, i.e. regional interhemispheric asymmetry of sleep depth. Similar unihemispheric sleep happens in some birds and marine mammals which is a protective mechanism to compensate for risks during sleep [8]. During pathological states such as epileptic seizure and Parkinson disease, some regions of brain are highly synchronized, indicating that the abnormally excessive synchronization is related to brain disorders [9]. In serious seizure events, the abnormal synchronization tends to spread to the whole cerebral cortex, leading to a global synchronization [10].

A question of fundamental interest to physical science and complex systems is how such diverse patterns are supported, shaped and constrained by the underlying interacting network. Advanced neuroimaging has revealed that there is a very complex cortico-cortical network underlying the dynamical and functional interaction among the distributed cortical regions, forming the brain connectome [11,12]. Brain network displays features like small world, communities, hierarchical and rich-club connectivity [11–13]. The study of the structure-function relationship has attracted great attention by investigating the interregional dynamical interactions from the method of functional connectivity (correlation of brain activities usually measured in functional magnetic resonance) and complex network measures [14,15]. It has been found that the structural and functional connectivity share many common graphic features [14,15]. Despite the advance made so far, how the underlying network architecture can support, shape and constrain various dynamical patterns at different brain states is still elusive and is a central challenge in network neuroscience [15].

A characteristic feature of the various brain dynamical patterns is that some nodes (cortical areas) are synchronized and the others are unsynchronized, i.e. a kind of coexistence of coherent and incoherent behaviors across different spatial scales in the whole brain system. In fact, the phenomenon of coexistence of coherent and incoherent states has been intensively studied in the last decade under the name chimera state (CS) for identical oscillators [16,17], including the neuron systems [18–20], experimental systems [21,22], and multiple CS [18,23]. Recently, it was reported that CS can even show up in real brain networks [24,25]. In particular, Bansal *et al*. studied CS in brain cortical network model at the level of nine cognitive subsystems formed by 76 cortical regions (network nodes) [26] and revealed a kind of cognitive CS due to stimulations to the cognitive subsystems. However, these studies of CS did not pay much attention to how CS is related to the underlying network properties.

Here, we construct a brain network from the real data of human cerebral cortex to study its collective behaviors. We focus on how the collective behaviors emerge from the interaction of neural populations on the underlying brain network. As each node of brain network represents a region of interest (ROI) with a large number of neurons, we use a neural mass model to describe the mean field activity of these neural populations. To go deep to the structure-dynamics relationship, we study the collective behaviors from both global and local regions, i.e. a rescaling or renormalization approach. We interestingly find that except disorder and synchronized states, there is a regime of CS in both levels, indicating a new feature of CS with multiple spatial scales. Especially, we find that a zero-correlation state of brain network in global level is very likely filled with abundant partially synchronized patterns in local level. To reveal the relationship between the activeness of each region in cognitive tasks and its connectome, we study the emergence of collective behaviors from the angle of synchronization and reveal a positive correlation between the synchronized fraction of a local region in phase diagram and the degree of connection symmetry of the region in the network.

Moreover, we use the concept of effective symmetry in the network to build structural and dynamical hierarchical trees from similarity in network connectivity and dynamical synchronization. We find a close matching between the structure and dynamical trees, which helps to explain the multiple brain rhythms observed in experiments and also reveals that the observed multiscaled CS patterns are inherent in the hierarchal structural clusters with different sizes. These clusters can be activated and recruited under different parameters to form diverse combinations of coherent-incoherent states. Thus, our work suggests a generic principle for complex brain network as a structure substrate to support diverse functional patterns under normal and abnormal conditions by activating different combinations of the inherent clusters offered by the complex network connectivity.

## RESULTS

Firstly, we construct a weighted brain network of cerebral cortex from the data of Refs. [11,12]. We notice from the data that there are $9$ isolated nodes without links, likely due to the limitation of diffusion tensor imaging method [11,12]. For convenience of discussion, we remove the $9$ isolated nodes in our modeling. The obtained network has $N = 989$ nodes (${N_r} = 496$ and ${N_l} = 493$ for the right and left hemispheres, respectively), each representing a ROI, and 17 865 links among all the nodes. The total nodes are grouped into $64$ functional regions following the cytoarchitecture and functional parcellation in [11,12], and those nodes in each of the $64$ cortical regions are numbered consecutively. The obtained $989 \times 989$ connection matrix $\{ {M_{\mathit{IJ}}}\} $ is weighted. Figs S1–S3 in supplementary information (SI) show the weighted connection matrices and partition of the $64$ cortical regions. Then, we let the dynamics of each node be represented by a neural mass model [27,28] describing the mean field activity of a neuronal population (see Methods). Results in the following are based on Eq. (2) in Methods with a constant time-delay $\tau $. The case of distributed $\tau $ will be discussed in Figs S6 and S7 of SI.

### Typical dynamical patterns and phase diagrams of order parameter

**Figure 1.**
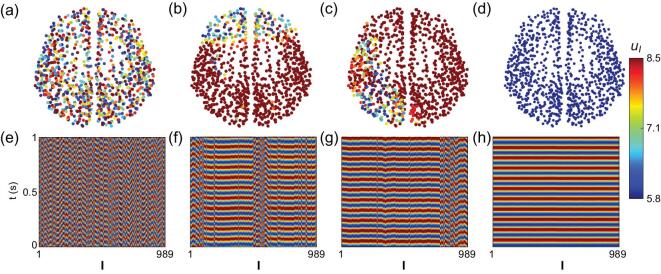
Typical dynamical patterns. (a-d): Snapshots of four typical patterns in the network of cerebral cortex with $N\ = \ 989$ nodes. (e-h): The spatiotemporal patterns corresponding to (a)–(d), respectively. $I\ = \ 1,\ \cdots ,496$ for right hemisphere and $I\ = \ 497,\ \cdots ,989$ for left hemisphere. The parameters are $c\ = \ 0.05$ and $\tau \ = \ 9\,{\mathit{ms}}$ in (a) and (e), $c\ = \ 0.025$ and $\tau \ = \ 16\,{\mathit{ms}}$ in (b) and (f), $c\ = \ 0.025$ and $\tau \ = \ 51\,{\mathit{ms}}$ in (c) and (g), and $c\ = \ 0.05$ and $\tau \ = \ 20\,{\mathit{ms}}$ in (d) and (h).

As in [27,28], we take the average potential ${u_I} = v_I^e\ - v_I^i$ from Eq. (2) to represent local field potential. Fig. 1 (a)–(d) shows the snapshots of four typical collective behaviors of the network for different pairs of coupling strength $c$ and time-delay $\tau $ and (e)-(h) shows their corresponding spatiotemporal patterns. Fig. 1 (a) and (e) shows that ${u_I}$ are largely randomly distributed, corresponding to an incoherent state. In Fig. 1 (b) and (f), the nodes are divided into different coherent groups, with some being unsynchronized with these groups, corresponding to a CS. Fig. 1 (c) and (g) gives the example that the right cerebral hemisphere is largely synchronized while many nodes in the left hemisphere are in an incoherent state, which resembles the phenomenon of unihemispheric sleep. Fig. 1 (d) and (h) shows a complete synchronization of the whole cerebral cortex, marking the large-scale synchronization in epileptic seizure.

**Figure 2.**
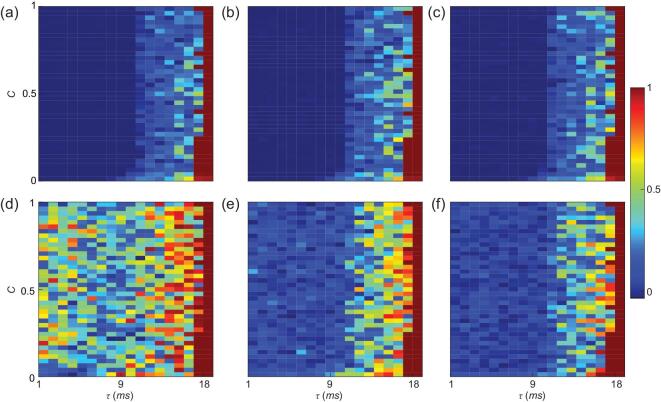
Phase diagram of the order parameter $R$ on the parameter space $\tau - c$ plane. (a), (b) and (c) represent the cases of whole brain network, right and left hemispheres, respectively, and (d)-(f) the cases of three typical cortical regions of 2 (rCAC, 4 ROIs), 38 (lFUS, 22 ROIs) and 7 (rIP, 28 ROI), respectively, randomly selected for demonstration.

Below we comprehensively explore the dynamical properties in the parameter space of $\tau - c$ plane through extensive numerical simulations and find diverse patterns. To quantify and distinguish these patterns, we adopt the order parameter $R$ from Eq. (3) in Methods. Fig. 2 shows the phase diagrams of $R$ on the parameter space $\tau - c$ plane, for different levels of the whole brain (a), hemispheres ((b) and (c)), and three typical brain regions ((d)-(f)).

Three features of Fig. 2 can be noticed: (i) The cases of Fig. 2(a)–(c) at global level are similar, thus the chance to find one hemisphere being synchronized and the other being incoherent at the same time must be located near the boundary between complete synchronization ($R$ ∼ 1) and un-synchronization ($R$ ∼ 0) in the phase space. (ii) Fig. 2(d)–(f) are largely different from each other in their un-synchronization regions ($R$ < 1). For example, the phase space for $\tau < 9$ is partially synchronized (0 < $R$ < 1) in Fig. 2(d) but incoherent ($R$ ∼ 0) in Fig. 2(f) and in between in Fig. 2(e). This local difference in dynamics is consistent with the observation that different cortical regions have been involved into different brain functions [9]. And (iii) the unsynchronized region of $R$ ∼ 0 in Fig. 2(a)–(c) with $\tau < 9$ is in contrast with the corresponding region of 0 < $R$ < 1 in Fig. 2(d), implying that a disordered state of $R$ ∼ 0 in global level of brain does not mean a complete disorder of the whole brain but a balanced state with abundant local synchronization patterns. This result can be further confirmed by other regions, see Fig. S5 in SI for the phase diagrams of $R$ on $\tau - c$ plane for all the $64$ cortical regions.

### Multi-scaled CS in brain network of cerebral cortex

By checking the dynamical behaviors of Fig. 2(d)–(f) in the regions with $0 < R < 1$ , we find that the dynamics of their oscillators typically consists of the coexistence of coherent and incoherent groups, suggesting that CS also appears at the local level. Thus, CS in brain network can be observed on both the global and local levels, which we call spatial *multi-scaled CS*.

**Figure 3.**
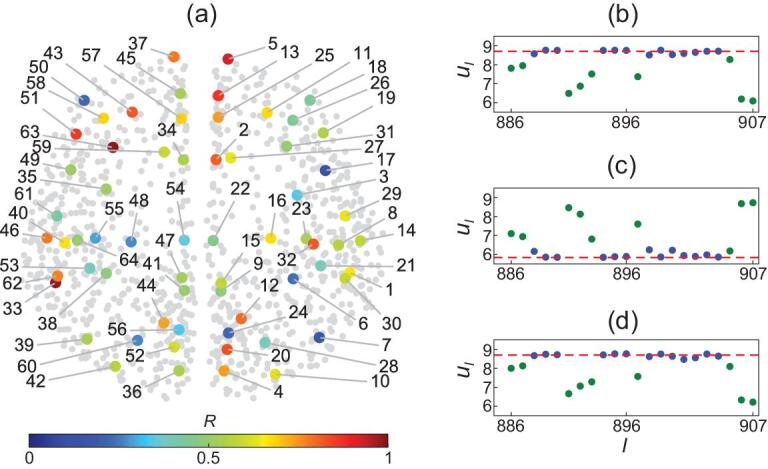
Spatial multi-scaled CS. (a) Local representation of $R$ for the case of $c\ = \ 0.075$ and $15\,{\mathit{ms}}$, where the color points with numbers represent the network of the $64$ local regions and the gray background points represent the network of $989$ nodes. The functional names of these $64$ local regions are given in Table S1 of SI. (b)–(d) show three arbitrary snapshots for the oscillators within the cortical region $38$ (lFUS) from (a), respectively, where the blue oscillators on the dotted lines represent the synchronized cluster which is stable.

To characterize the CS of local level, we go to the scaled network of the $64$ brain cortical regions [11,12], where a region-$i$ consists of ${n_i}$ oscillators. Considering each region as a node, the scaled network will have $64$ nodes, where the physical position of each node will be the average of the positions of all its ${n_i}$ nodes. Fig. 3(a) shows the position distribution of these $64$ nodes in human brain network where the numbers are the index of these nodes and their functional names are given in Table S1 of SI. We also keep the original $989$ nodes in Fig. 3(a) as the gray background, for visualization effect. Now, we calculate the local order parameter $R$ for the ${n_i}$ nodes in each local region. Take the case of $c\ = \ 0.075$ and $\tau \ = \ 15\,{\mathit{ms}}$ as an example, which has a small $R$ at the global level. The different colors of nodes in Fig. 3(a) show their values of $R$*.* We see that the degree of synchronization differs across cortical regions in the whole brain. To confirm the feature of CS in local level, Fig. 3(b)–(d) shows the snapshots of those oscillators within the cortical region $38$ (lFUS) from (a) for three arbitrary moments, respectively. We see that the oscillators on the dotted lines represent the synchronized clusters and others unsynchronized, confirming the coexistence of stable synchronized cluster with incoherent oscillators, i.e. a CS within the region $38$ (lFUS). We have observed the similar phenomenon at other nodes of Fig. 3(a) with $0 < R < 1$ and also found that it is quite general for different parameters $( {\tau ,\! c} )$ (e.g. see Fig. S4 in SI, corresponding to Fig. 1(b)).

### Dependence of node's activeness on local structure of brain network

**Figure 4.**
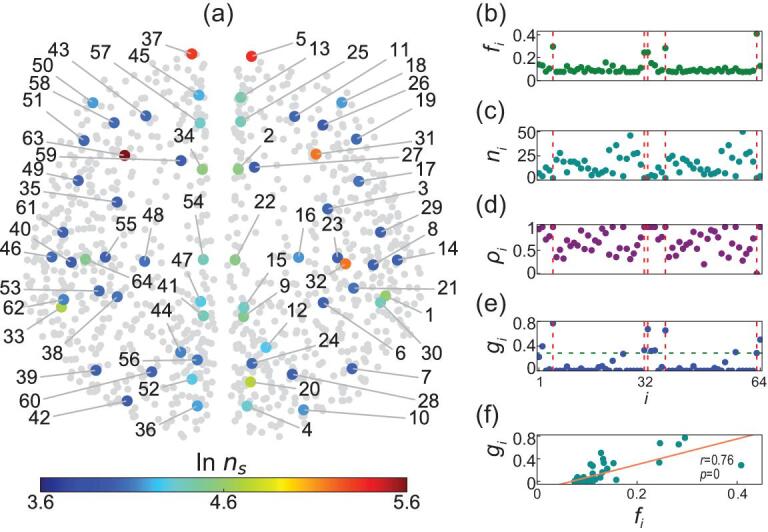
Node's activeness and its dependence on local structure of brain network. (a) Number of synchronized points ${n_s}$ with $R > 0.9$ in the phase diagram of $\tau - c$ plane, where the coupling range $0\sim1$ is divided into $40$ points, and the time-delay range $1\sim 18\,{\mathit{ms}}$ is divided into $18$ points. (b)–(e) respectively show ${f_i}$, ${n_i}$, ${\rho _i}$, and ${g_i}$ for the 64 regions. (f) ${g_i}$ versus ${f_i}\ $with Pearson correlation coefficient r = 0.76 and *p*-value *p* = 0.

Then, a key question is how the CS of these $64$ local regions are related to their brain functions. To figure out the answer, we show the local $R$ of all the $64$ cortical regions for a few typical sets of c and τ in the Table S1 and find that the individual regions have different preferences to show the states of disorder, CS, and synchronization. This result is interesting as it confirms that different local regions of brain have different activeness to potentially support their heterogeneous roles in performing certain tasks and cognitive functions such as pattern recognition, function approximation, and data processing etc. To quantitatively measure this activeness, we sample the phase diagram of each local region (i.e. Fig. 2(d)–(f) and Fig. S5) by $\delta \tau \ = \ 1\,{\mathit{ms}}$ in the range of $1\sim18$ and $\delta c\ = \ 0.025$ in the range of $0\sim1$ , i.e. total $18{\rm{\ }} \times {\rm{\ }}40{\rm{\ }} = {\rm{\ }}720$ phase points in the $\tau - c$ plane. Then, we count the number of phase points with *R* > 0.9 from the phase diagram of each local region and denote it as ${n_s}$. Fig. 4(a) shows the results where $\ln {n_s}$ ranges from $3.6$ to $5.6$, i.e. ${n_s}$ ranges from $50$ to $294$. Three features can be found from this figure: (i) There are a few regions with higher ${n_s}$ such as the regions $5,{\rm{\ }}37,{\rm{\ }}31,{\rm{\ }}63$ and $32$. (ii) Most of the regions with middle values of ${n_s}$ are distributed along the middle lines separating the two hemispheres. (iii) And most of the regions with lower values of ${n_s}$ are distributed away or far away from the middle lines. For convenience, we let ${f_i} = {n_s}/720$ represent the fraction ${f_i}$ for each region-$i$ to take $R > 0.9$ in the $\tau - c$ phase plane, representing the activeness of the region in synchronization dynamics.

**Figure 5.**
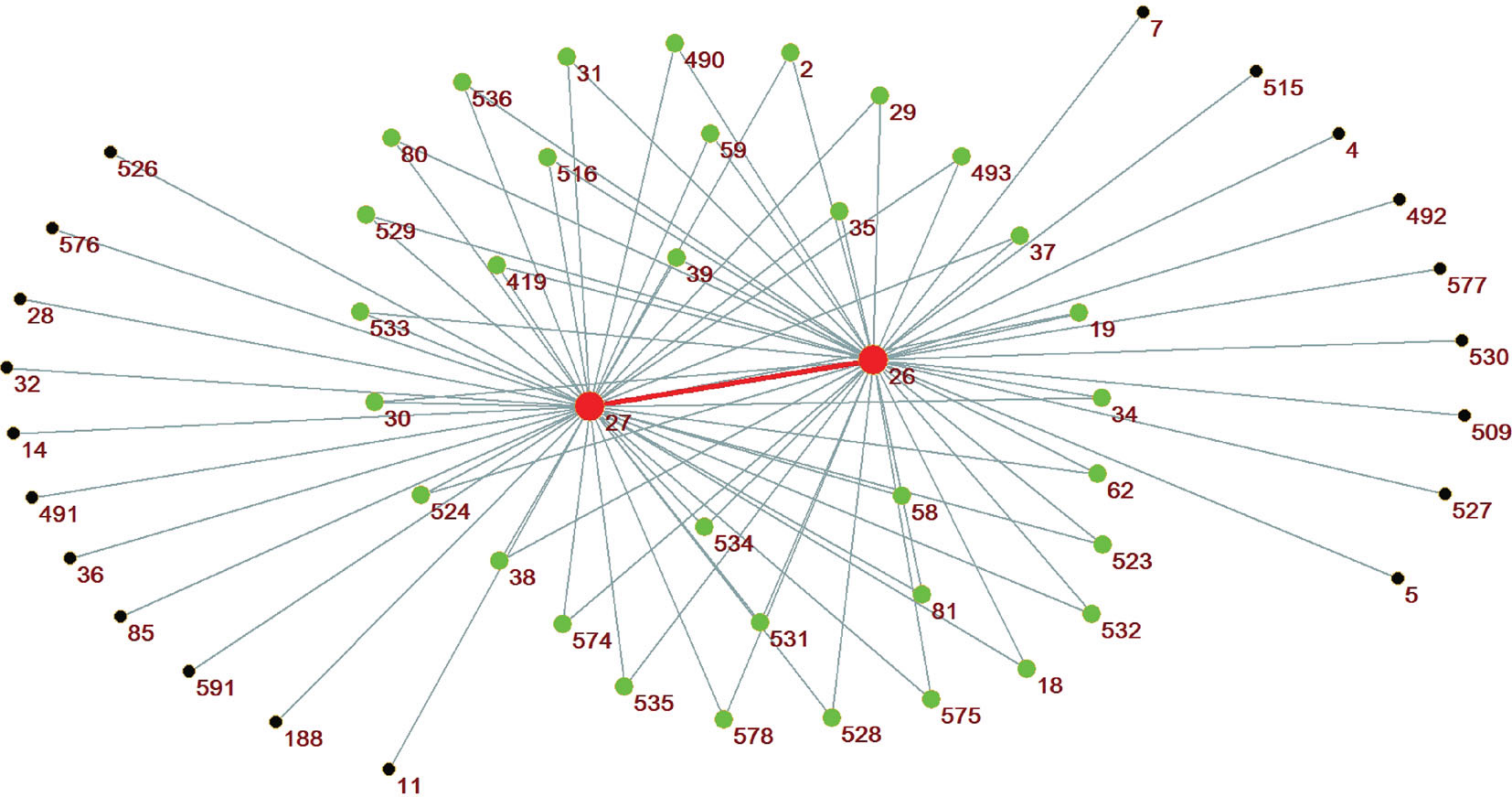
Connection structure and symmetry of the region-5. The region-5 contains only two oscillators (ROIs 26 and 27) where the red line is the only intra-link and the other links are the out-links. The common nodes are shown in the central part with green color.

To understand the underlying mechanism of these three features or how the local topologies of nodes influence their behaviors, we recall the recent findings of cluster synchronization that the oscillators of network will be automatically evolved into different synchronized clusters but the oscillators in different clusters are not synchronized with each other [29,30]. It was revealed that network symmetry is the necessary condition for synchronized clusters [31,32]. It was also reported that the synchronized cluster may not directly result from the network symmetry, but due to the same total amounts of inputs received from their neighboring nodes [33]. Thus, we may conclude from these findings that more symmetry among a cluster of oscillators implies stronger synchronization among them. In our case, we consider the ${n_i}$ oscillators of region-$i$ as a cluster. Its symmetry comes from two aspects. One is the symmetry from the intra-links among the ${n_i}$ oscillators, and the other is the symmetry from the out-links of the ${n_i}$ oscillators. For the first aspect, a complete graph, where each oscillator has an intra-degree $k_i^{in} = {n_i} - 1,\ $corresponds to a perfect symmetry since each pair of oscillators have completely common network neighbors$.$ When the graph is not complete, we let ${\rho _i} = \displaystyle\frac{{{n_i}\langle {k_i^{in}} \rangle }}{{{n_i}( {{n_i} - 1} )}}\ $be the intra-connection density where $\langle {k_i^{in}} \rangle $ is the average intra-degree and ${n_i}( {{n_i} - 1} )$ is the maximum of possible intra-links within the same region-$i$. Then, a larger connection density ${\rho _i}$ corresponds to a stronger symmetry of intra-links. For the second aspect, the out-links of the ${n_i}$ oscillators will go to different oscillators of the neighboring regions of region-$\ i$. Consider a specific case where each of the ${n_i}$ oscillators has only one out-link and all the ${n_i}$ out-links go to the same oscillator-j in another region. In this case, the oscillator-j is a center hub while the ${n_i}$ oscillators are the leaf nodes, i.e. a star network. It has been revealed that for the star network, there is a remote synchronization among the leaf nodes of a hub but not synchronized with the hub [34]. This result has been recently extended to the brain network [35]. As the leaf nodes are symmetric around the hub, we may conclude that the existence of the common hub oscillator-j represents the symmetry of out links. Thus, more common hub oscillators imply stronger symmetry of out links. We let ${g_i} = \frac{{n_i^{comm}}}{{ < k_i^{out} > }}\ $be the out-links symmetry of the ${n_i}$ oscillators where $n_i^{comm}$ represents the number of the common hub oscillators and $ < k_i^{out} > $ represents the average out-links of the ${n_i}$ oscillators, i.e. the average out degree. A larger ${g_i}$ means a stronger out-links symmetry. For example, Fig. 5 shows the case of the region-5 with ${n_5} = \ 2$ in Fig. 4(a) where the red line is the only intra-link, the other links are the out-link, and the green nodes are the common hub oscillators. After simple calculation we obtain ${\rho _5} = \ 1$ and ${g_5} = \ 0.767$. Doing the same calculation for all the 64 regions, Fig. 4(b)–(e) respectively show ${f_i}$, ${n_i}$, ${\rho _i}$, and ${g_i}$ for all the 64 regions. Several features can be noticed for the five nodes $5,{\rm{\ }}37,{\rm{\ }}31,{\rm{\ }}63$ and $32$ with higher ${n_s}$ in Fig. 4(a). From Fig. 4(b)–(e) we see that all the five nodes have ${f_i} > 0.2$ in (b), a small ${n_i}\ $in (c), a larger ${\rho _i}\ $in (d), and a larger ${g_i}\ $in (e). In sum, the oscillators in all the five nodes have stronger symmetries as they all have both larger ${\rho _i}\ $and larger ${g_i}$, which explains why we have observed the larger ${f_i}$ or ${n_s}$ for the five nodes. To check the statistical significance of these results, we have calculated the correlation between ${f_i}$ and ${g_i}$ and its *P*-value, see Fig. 4(f). We find that its Pearson correlation coefficient is r = 0.76 and *P*-value is *P* = 0.

On the other hand, we find that the value of ${n_s}$ is closely related to the node's activeness in brain functions. For example, the five nodes $5,{\rm{\ }}37,{\rm{\ }}31,{\rm{\ }}63,$ and $32$ with the largest values of ${n_s}$ in Fig. 4(a) represent the brain regions rFP, lFP, rTP, lTP, and rTT, respectively, see Table S1. It is well known that all these regions take key roles in the aspects of cognitive, memory, behavior, and auditory processing etc. [36]. We also notice that each of these five regions contains only a few oscillators ( ${n_i}$ = 2 for the regions of rFP, lFP, rTP and lTP and ${n_i}$ = 3 for rTT) and thus mainly takes the role of connecting other regions, i.e. the function of signal transmission [36]. These results show robustness to distributed time delays (see Figs S6 and S7 in SI).

### Hierarchy trees of both anatomical and functional networks

To further explore how the network structure can support the multiscaled dynamics, we would like to extend the above concept of *hidden dynamical symmetry* in the network [31–33,37–39]. Two nodes $\mathit{l}$ and $\mathit{l}^{\prime}$ are dynamically symmetrical if they have exactly the same connection neighbors $nn$ in the network so that their dynamical evolutions are identical and their states can be in principle completely synchronized, i.e.

(1) \begin{eqnarray*} {\dot{x}_\mathit{l}} &=& \ {{\bf f}}\left( {{x_\mathit{l}}} \right) + \mathop \sum \limits_{k \in nn} {M_{\mathit{l}k}}{{\bf h}}\left( {{x_k}} \right),\ {\dot{x}_{\mathit{l}^{\prime}}} = \ {{\bf f}}\left( {{x_{\mathit{l}^{\prime}}}} \right)\nonumber\\ && +\, \mathop \sum \limits_{k \in nn} {M_{\mathit{l}^{\prime}k}}{{\bf h}}\left( {{x_k}} \right). \end{eqnarray*}

However, in the real brain networks, dynamical symmetry may not be perfect, but only be effective, so that two nodes may not have exactly the same network neighbors, but may share a portion of common neighbors. Higher portion of common neighbors will give stronger common driving signals to the two nodes and will likely make stronger synchronization between them. Thus, it is possible to predict synchronization patterns from the inherent patterns of effective symmetry in the network connectivity. Based on a weighted connectivity matrix **W**, with ${W_{\mathit{l}\mathit{l}^{\prime}}}$ being the input connection from node $\mathit{l}^{\prime}$ to node $\mathit{l}$ , let ${K_\mathit{l}}$ be the total weight of node-$\mathit{l}$, i.e. ${K_\mathit{l}} = \mathop \sum \nolimits_k {W_{\mathit{lk}}}\ $from all other nodes. The shared weight for nodes $\mathit{l}$ and $\mathit{l}^{\prime}$ from a common neighbor $n$ is $O_{\mathit{ll}^{\prime}}^n = \ {\mathit{min}}( {{W_{\mathit{ln}}},\ {W_{\mathit{l}^{\prime}n}}} )$, thus $\mathop \sum \nolimits_n O_{\mathit{ll}^{\prime}}^n\ $represents the total shared weight from all the common neighbors of the two nodes. We can quantify the effective dynamical symmetry by the similarity of connectivity between two nodes $\mathit{l}$ and $\mathit{l}^{\prime}$ as ${S_{\mathit{ll}^{\prime}}} = \ ( {\mathop \sum \nolimits_n O_{\mathit{ll}^{\prime}}^n} )/( {{K_\mathit{l}} + {K_{\mathit{l}^{\prime}}} - \mathop \sum \nolimits_n O_{\mathit{ll}^{\prime}}^n} )$ , i.e. the ratio of total shared weight from all the common neighbors to the union of connection weights of the two nodes in the network. ${S_{\mathit{ll}^{\prime}}} = \ 1$ for ideal symmetry if the neighbors are completely identical or ${S_{\mathit{ll}^{\prime}}} = \ 0$ if there is no sharing of any common neighbor at all. In previous work [28,40], this degree of symmetry was called matching index and used to obtain the hierarchy tree of anatomical network [40], by performing hieratical clustering analysis of the dissimilarity $1 - {S_{\mathit{l}\mathit{l}^{\prime}}}$. Below we focus our attention to analyzing the relationship between the hierarchical organizations of the effective symmetry in anatomical connectivity and the dynamical clustering of spatial multiscaled CS. We will study the structure-dynamics relationship from both the regional level with $64$ cortical regions and the ROI level with $989$ nodes.

**Figure 6.**
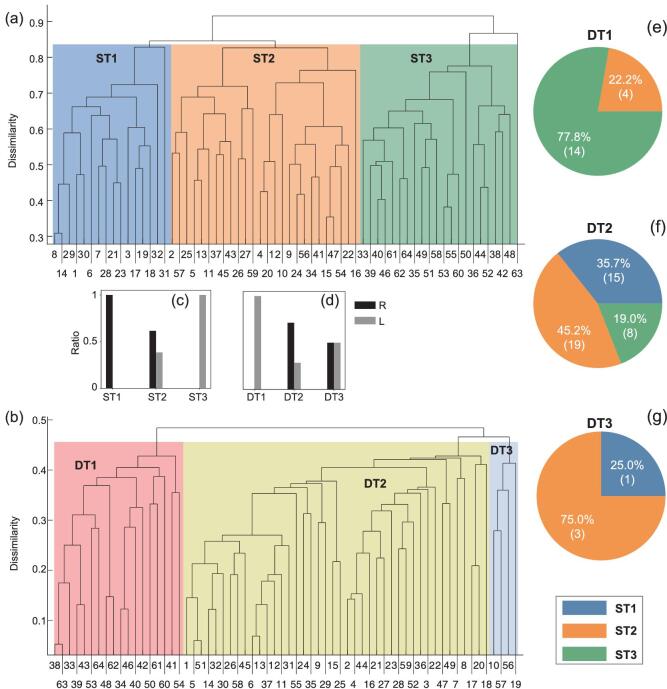
Relationship between hierarchal clusters of anatomical network and dynamical synchronization at the level of $64$ cortical regions. (a) Hierarchy tree of anatomical network with three branches ST1, ST2, and ST3 by taking ${y_{th}} \approx 0.84$. (b) Hierarchy tree of functional network with three branches DT1, DT2, and DT3 for $c\ = \ 0.075$ and $\tau \ = \ 15\,{\mathit{ms}}$ by taking ${y_{th}} \approx 0.45$ . (c) Fractions of nodes from right and left hemispheres in ST1- ST3 of (a). (d) Fractions of nodes from right and left hemispheres in DT1-DT3 of (b). (e)-(g) show the fractions (number) of nodes in DT1-D3 of (b) coming from ST1-ST3 in (a), respectively.

For the coarse-grained network of $64$ cortical regions, we consider two cortical regions $\mathit{l}$ and $\mathit{l}^{\prime}$ be connected if there is at least one link between their nodes. The weight ${W_{\mathit{l}\mathit{l}^{\prime}}}$ of this inter-regional connection will be the average weight for all those links between ROIs in the two regions (see Fig. S3 in SI). Figure 6(a) shows the result of hierarchical tree of the anatomical network where $x$ axis is the $64$ cortical regions and$\ y$ axis is the dissimilarity. This hierarchical tree clearly displays connectivity clusters across multiple levels, which can be obtained from the sub-trees (branches) at different thresholds ${y_{th}}$. Here we classify it into three structural trees ST1, ST2, and ST3, respectively, by taking ${y_{th}} \approx 0.84$ . While ST1 and ST3 are solely from the right and left hemispheres, respectively, ST2 is the combinations of the two hemispheres (see Fig. 6(c)). We notice from Fig. 6(a) that the isolate $63$ does not belong to the three trees, which is consistent with its role of relay node. Similar situations will happen for other relay nodes when different ${y_{th}}$ are taken.

We now obtain the hierarchy tree of dynamical interactions (functional network) from the neural mass model in two steps. (i) We calculate the pairwise order parameter ${R_{\mathit{IJ}}}$ from Eq. (4) for all the pairs of nodes *I* and *J* for all the $989$ nodes. (ii) We calculate the average value for all those functional links ${R_{\mathit{IJ}}}$ between the two cortical regions $\mathit{l}$ and $\mathit{l}^{\prime}$ as the dynamical similarity $S_{\mathit{l}\mathit{l}^{\prime}{\rm{\ }}}^F$ and obtain the hierarchal tree using dynamical dissimilarity $1 - S_{\mathit{l}\mathit{l}^{\prime}{\rm{\ }}}^F\!$. Figure 6(b) shows the hierarchy tree of the functional network for $c\ = \ 0.075$ and $\tau \ = \ 15$ by taking ${y_{th}} \approx 0.45$, which is also divided into three branches, named as dynamical trees DT1, DT2, and DT3, respectively. The ratios how each dynamical branch comes from the right and left hemispheres in Fig. 6(d) show that DT1 is solely from the left hemisphere, while DT2 and DT3 are from both the left and right hemispheres. These results are well consistent with the observations in Figs 1 and 3.

Now, we investigate the relationship between structural and dynamical clusters (branches) in Fig. 6(a) and (b) by examining how the dynamical trees in Fig. 6(b) are contributed by the underlying structural tree in Fig. 6(a). Figure 6(e)–(g) shows the results where DT1 is mainly from ST3, DT2 is a rich combination of all the structural branches, with ST2 giving the maximal contribution, and DT3 is dominated by ST2. Overall, the left hemisphere is divided by all the three dynamical clusters DT1-DT3, suggesting that the left hemisphere appears to be more segregated in this dynamics state. Similar matching relationship holds when considering fewer branches, where ST1 and ST2 are merged to a large structural branch containing the whole right hemisphere and part of the left hemisphere and DT2 and DT3 are merged to a larger dynamical cluster (see Fig. S8). Similar relationship between dynamical and structural trees also holds for other parameters.

**Figure 7.**
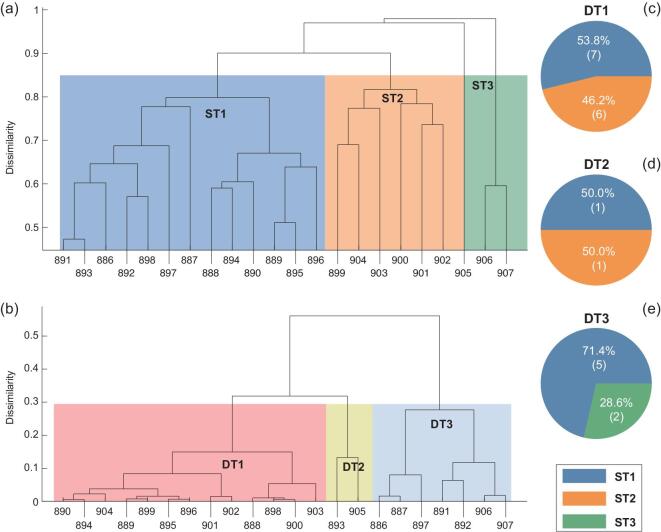
Matching of hierarchal cluster between anatomical network and dynamical synchronization at the level of $989$ nodes: the hierarchy tree of the cortical region $38$ (lFUS). (a) Hierarchy tree of anatomical network with three branches ST1, ST2 and ST3. (b) Hierarchy tree of functional network with three branches DT1-DT3, where the parameters are taken as $c\ = \ 0.075$ and $15\, ms$. (c)-(e) represent the fractions of those lFUS nodes in DT1-DT3 of (b) from those lFUS nodes in ST1, ST2 and ST3 in (a), respectively.

The multiscaled CS can be further elucidated by investigating the anatomical and functional networks level with $989$ ROIs. As it is not readable to plot the $989$ nodes in a single figure, we here only take the branch containing most of the cortical region $38$ (lFUS) with $22$ nodes as an illustration example. Figure 7(a) shows its hierarchical tree of anatomical network with three structural branches of ST1, ST2 and ST3. It is easy to see that the hierarchical tree of Fig. 7(a) is similar to that of Fig. 6(a), indicating a scaling invariance. This property is consistent with the observation that cortical network connectivity is cost-efficient [40], with strong projections in spatial neighborhood and decaying exponentially with distance.

Figure 7(b) shows the hierarchical tree of functional network of the cortical region $38$ (lFUS). Similar to Fig. 6(e)–(g), we also find that the majority of each of the dynamical trees DT1-DT3 always comes from two structural branch of ST1, ST2, and ST3 (Fig. 7(c)–(e)), confirming again the close relationship between the structural and functional networks. This matching is similar for other cortical regions displaying CS.

## DISCUSSION

In this work, we study the principle how complex brain network can support diverse co-existence of coherent and incoherent dynamics patterns in the framework of CS of identical oscillators in networks. Our intensive numerical simulations on biologically plausible neural mass models, ideally assumed to be identical in different ROIs, have shown that the highly complex and heterogeneous brain network can support spatial multiscaled CS at different coupling and delay parameters. We reveal that the fundamental principle lies in the inherent effective symmetry in the complex brain structural networks, that allows the formation of hierarchical synchronization clusters across multiple scales, and such clusters can be activated and manifested under different dynamical parameters.

The multiscaled CS suggested that there is a similar organization principle underlying the hierarchy of the brain network. Intuitively, we may make a rescaling/renormalization process to change the size of brain network where a few nodes are combined to form a larger one. If we intentionally let the synchronized, CS and unsynchronized nodes of Fig. 3(a) to combine to form new nodes, respectively, we will have similar dynamics on different scales. Continuing this process, we will finally go to the global level where all the oscillators/nodes are considered as a unit. We have confirmed this renormalization process in numerical simulations. As an example, Fig. 2(f), (b), and (a) shows the phase diagrams of $R$ for the region 7 (rIP), right hemisphere, and whole brain network on the parameter space $\tau - c$ plane , respectively. We see that these three panels with different size are similar to each other, confirming the feature of spatial multi-scaled *CS*.

The results in Figs 6 and 7 comparing the hierarchical clustering in structural and functional connectivity suggested that the underlying network can provide substrate to support diverse dynamical patterns through the organization of hierarchical clusters across multiple scales due to effective symmetry, and these inherent structural clusters can dominate dynamical clusters or provide flexible segregation-integration to form rich dynamical combinations. Generally, the anatomical brain network in adults does not have a significant change within short periods. Thus, the implementation of brain activities and functions could be manipulated by the coupling strength (neurotransmitters) and other possible physiological parameters. The variation of coupling strength represents the activation or inactivation of links, while different time-delays correspond to long or short links, respectively. When the brains stay in different states, such as in wakefulness or sleep, the effective coupling could be manipulated to select and activate different inherent states and their combinations. Therefore, the abundant combinations between $\tau $ and $c$ potentially guarantee the diversity of brain patterns.

The hierarchical clustering and multisaled CS could also contribute to the formation of multiple rhythms in brain activities. In a multiscale network system, the spatial scales and temporal scales are inherently related based on the eigenmode theory [41,42]. Indeed, it has been shown that brain temporal rhythms are closely coupled with spatial scales, with faster oscillations in local scales and slower oscillations in broader scales [43,44]. For example, during sleep, slow oscillations can emerge to generate large-scale synchronized states [5,6] recognized as slow wave sleep. In the current work, we have shown that the multiscaled CS is closely related with the underlying hierarchical spatial clusters that are inherent in the network structure. Considering the interactions within and between such clusters, their different sizes of feedback loops represent different rhythms of neural activities. When needed for specific brain functions, these rhythms/clusters may be activated and recruited under different parameters to form diverse combinations of coherent-incoherent states, which might explain the multiple brain rhythms observed in experiments. Thus, these clusters of different sizes provide a scalable backbone for multiscaled CS patterns and suggest a generic principle for complex brain network as a structure substrate to support diverse functional patterns under normal and abnormal conditions. In this sense, we may consider the multiscale feature of chimera state as one potential mechanism for the multiple brain rhythms observed in EEG or any other electrophysiological brain measurements, but not the only one. The multiple brain rhythms may also come from other factors such as the external stimulations and the task state or resting state etc.

Our results showed that the tendency to form coherent synchronization clusters in different brain regions is heterogeneous due to nonuniform inherent symmetry property of the underlying brain network. The synchronization of different regions could be closely related to the brain functions and disorders. Especially, an abnormal synchronization is the mark of epilepsy. There are different types of epilepsy. Sometimes there are focal areas in the frontal lobe or temporal lobe, and sometimes enhanced synchronization is more broadly distributed. For temporal lobe epilepsy (the type where surgery is often the only option), thalamus, amygdala, and hippocampus are involved [45]. Interestingly, this phenomenon is consistent with our results, i.e. the five regions 5 (rFP), 37 (lFP), 31 (rTP), 63 (lTP) and 32 (rTT) with the largest values of *ns* in Fig. 4(a). The regions 31 (rTP) and 63 (lTP) are just the right and left temporal lobes, while the regions 5 (rFP) and 37 (lFP) are just the right and left frontal poles, i.e. the tops of frontal lobes. This striking correspondence suggested that epilepsy may have an anatomical foundation rooted in the symmetry property of the brain network. This correspondence also suggests that the hierarchal symmetry in the brain network as revealed in this work may offer a principle understanding of various cognitive processes in the brain, which deserves further theoretical and experimental investigations.

In the current work, we have considered identical neural oscillators for different ROIs. Recent neuroanatomical studies have shown that the local cortical regions are not uniform in its thickness, neuronal density, spine density, myelination contents and gene expressions [46,47], thus the internal dynamics of ROI is supposed to be heterogeneous too, e.g. with different time scales [48,49]. In our modeling framework at the level of ROI, such nodal heterogeneity in the oscillators is supposed to induce additional diversity and to enlarge the range of complexity of the neural dynamics, as enhanced or even optimized dynamical complexity is desirable for functional requirements, e.g. to achieve a balance of segregation and integration across different scales [41]. If we go into the microscopic scales of interacting neurons by synaptic coupling, such heterogeneity in local circuits in ROIs also corresponds to heterogeneous network connectivity among the neurons, and is expected to induce more levels of effective symmetry and synchronized firing clusters within ROI, again supporting the notion of more complex dynamics for efficient information coding and processing.

Another limitation of this study is that the considered brain network is only in the level of cortical regions, i.e. about 1000 nodes. In this level, the subcortical structures, such as amygdala, hippocampus and thalamus, have not been included in the model, but they are network hubs and are supposed to play a crucial role in both functionally desirable synchronization and pathological brain dynamics. Recently, we notice a brain network of higher resolution with 50 000 nodes [50], which is scale-invariant across topological scales [51]. Such finer resolution brain network would better reflect the heterogeneity in local connectivity. We will study such finer resolution brain network in the future.

## CONCLUSION

In sum, we have shown that coupled neural mass oscillators on human cerebral cortex network can display spatial multiscaled CS, i.e. both global and local levels. A global state of $R\ \sim\ 0$ may correspond to a variety of local patterns with $1 > R > 0$ , indicating that the CS on larger scales can be considered as a rescaling of those CS on small scales. Further, we have shown that the effective symmetry in the network connectivity forms hierarchical clusters in the network which can potentially form multi-scaled dynamical clusters, but the recruitment of these inherent structural clusters to form rich dynamical clusters depends on the coupling strength and delay parameters in the current model. These findings elucidate a generic principle underlying the structure-function relationship in the brain, namely the underlying complex cortical network can support diverse brain dynamical patterns by activating different combinations of the hidden inherent clusters under different normal or abnormal physiological and psychological conditions. It is plausible to expect that the heterogeneity in local neural circuits [46] could bring in additional rich dynamical diversity for efficient functioning, while the counter-intuitive results that the heterogeneity in oscillators or connectivity may compensate the imperfect symmetry to enhance synchronization [31] could also play a role in brain functioning, which are interesting lines of research in the future.

## METHODS

### Dynamical equations

As each ROI of the real brain network contains an ensemble of excitatory and inhibitory neurons, we model its dynamics by a neural mass model [27,28] describing the mean field activity of a neuronal population. This low-dimensional model with biologically plausible interactions between excitatory and inhibitory neural populations can generate oscillations in the alpha band (∼ 10 Hz) and is used to represent resting brain states [52]. Increasing evidences show that the local circuits in the cortical regions are not identical [46,47], but display heterogeneity in neuronal density and spine density etc. However, modeling the regions with simplified assumption of identical neural mass oscillators allows us to focus on the effect of the underlying network architecture on the dynamical patterns. The dynamical equations of identical neural mass oscillators coupled by the underlying cortical network read as

(2) \begin{eqnarray*} \ddot{v}_I^p &=& \ Aaf\left( {v_I^e - v_I^i} \right) - 2a\dot{v}_I^p - {a^2}v_I^p\! ,\nonumber\\ \ddot{v}_I^i &=& Bb{C_4}f({C_3}v_I^P) - 2b\dot{v}_I^i - {b^2}v_I^i,\nonumber\\ \ddot{v}_I^e &=& Aa\Big[{C_2}f\left({C_1}v_I^p\right) + {p_I} + \frac{c}{{{\lambda _I}}}\sum\nolimits_{J = 1}^N {M_{\mathit{IJf}}}\nonumber\\ &&{\left(v_J^e(t - \tau ) - v_I^e\right)} \Big] - 2a\dot{v}_I^e - {a^2}v_I^e, \end{eqnarray*}

where $I\ = \ 1,\ \cdots ,\ N\ $,$\ v_I^p$, $v_I^i$ and $v_I^e$ are the post-synaptic membrane potentials for three subpopulations (pyramidal neurons, inhibitory and excitatory interneurons) of the node-$I\! $. The sigmoid function $f( v )$ converts the average membrane potential into an average pulse density of action potentials (spikes), which propagate among subpopulations within each node and between nodes through synaptic coupling. ${M_{\mathit{IJ}}}$ is the coupling matrix with the real connection weights from the data of Refs. [11,12]. The coupling strength $c$ is normalized by the mean intensity ${\lambda _I}$ across the nodes, where ${\lambda _I} = \mathop \sum \nolimits_J^N {M_{\mathit{IJ}}}$ is the total input weight to node-$I$. The parameters $A$ and $B$ represent the average synaptic gains, and $1/a$ and $1/b$ are the average dendritic-membrane time constants. ${C_1}$ and ${C_2}$ , ${C_3}$ and ${C_4}$ are the average number of synaptic contacts among the subpopulations. A more detailed interpretation and the standard parameter values of this model can be found in [27,28]. In this work, we follow Ref. [28] to take the parameters as $\mathit{cc}\ = \ 135$, ${C_1} = \ \mathit{cc}$, ${C_2} = \ 0.8\mathit{cc}$, ${C_3} = \ 0.25\mathit{cc}$, ${C_4} = \ 0.25\mathit{cc}$, $A\ = \ 3.25$, $B\ = \ 22$, $a\ = \ 100$, $b\ = \ 50$, and ${p_I} = \ 180$. The sigmoid function takes the form $( v ) = \ 2{e_0}/( {1 + {e^{r( {{v_0} - v} )}}} )$, where ${v_0}$ is the postsynaptic potential corresponding to a firing rate of ${e_0}$, and $r$ is the steepness of the activation, with the parameters as ${v_0} = \ 6$, ${e_0} = \ 2.5$, and $r\ $ = 0.56 as in Refs. [28,29]. $\tau $ is the time-delay for interregional signal transmission, assumed to be common for different links. This model setting of delay is certainly simplified as the conduction speed depends on whether the synapses are myelinated or not and on the length of the fibers and differs across pieces [53]. To date, complete information of conduction delays of the brain network is not available. The more realistic case of distributed $\tau $ according to distance between ROIs is discussed in Figs S6 and S7 in SI.

In the study of CS [16,17], the coupling delay $\tau $ is often considered as a tunable parameter. Below we aim to demonstrate that under different parameter settings $\tau - c$, various CS patterns can emerge from the inherent effective symmetry in the underlying network, though we cannot simply claim that these parameters are the actual biological reasons for the formation of a particular pattern in the real brain.

### Order parameters

To quantify and distinguish the patterns, we adopt the order parameter $R$ in the form

(3) \begin{equation*}R\ {e^{i\phi }} = \frac{1}{{{N_i}}}\ \mathop \sum \limits_{I\ = \ 1}^{{N_i}} {e^{i{\theta _I}}}\! ,\end{equation*}

where $R$ characterizes phase coherence, $\phi $ the average phase, $\theta $ the phase of oscillator, and ${N_i}$ is the number of coupled oscillators to be examined. The phase variable of a general nonlinear oscillator not necessarily having a well-defined rotational center can be obtained based on the general idea of the curvature [54], namely ${\theta _I} = \ {\rm{arctan}}( {\dot{v}_I^i/\dot{v}_I^e} )$ in our system. We consider cases of both the global and local levels. In the global level, ${N_i}$ will be $N\ = \ 989$ for the whole brain network, and ${N_r} = \ 496$ and ${N_l} = \ 493$ for the right and left hemispheres, respectively. In the local level such as within each of the $64$ brain cortical regions, ${N_i}$ will be the number ${n_i}$ of nodes in the region-$i$.

We can also introduce another order parameter ${R_{\mathit{IJ}}}$ to describe the correlation between two connected nodes, i.e. the pairwise order parameter, defined as

(4) \begin{equation*} {R_{\mathit{IJ}}} = \ \left| {\mathop {\lim }\limits_{T \to \infty } \frac{1}{T}\mathop \int \nolimits_t^{t + T} {e^{i\left[ {{\theta _I}\left( t \right) - {\theta _J}\left( t \right)} \right]}}dt} \right|, \end{equation*}

where ${\rm{T}}$ is the time window to measure synchronization. Thus, ${R_{\mathit{IJ}}}$ represents the correlation between nodes $I$ and $J$ for all the 989 nodes.

## Supplementary Material

nwaa125_Supplemental_File
