# Supplementary material for: Spatial multi-scaled chimera states of cerebral cortex network and its inherent structure-dynamics relationship in human brain
Source: Natl Sci Rev. 2020 Jun 5;8(1):nwaa125. doi: 10.1093/nsr/nwaa125 (PMC8288421; doi:10.1093/nsr/nwaa125)
Supplement: nwaa125_Supplemental_File [file nwaa125_supplemental_file.pdf]

H2: Supplementary Materials

A. Topology of the cerebral cortex network

In this work, we study how chimera states with multiple scales can emerge from the underlying structural substrates of human cortical network, which can provide new insights into the generic dynamical network principle underlying brain structure-function relationship. For this purpose, we use the data of Ref. [1, 2] to construct a network of cerebral cortex. In this data, the cerebral cortex was divided into relatively uniform 998 regions of interests (ROIs) with each representing a network node, and the connections in all possible pairs of 998 ROIs were measured noninvasively by using diffusion spectrum imaging (DSI). In this way, a connection between two ROIs was derived from the number of fibers found by the tractography algorithm, which results in 17865 connections and 9 isolated nodes without detected fibers due to resolution limitation of DSI. Furthermore, the cerebral cortex can be parcellated into 66 functional regions [1, 2]. In our work, we here remove the 9 isolated nodes, leaving $N = 989$ nodes, with $N_r = 496$ nodes in the right hemisphere and $N_l = 493$ nodes in the left hemisphere. The number of cortical regions covered by these nodes is also reduced to 64. The obtained 989×989 connection matrix is actually weighted, with the connection weights representing the fiber density between the connected nodes. Fig. S1 shows the weighted connection matrix, where the points represent the presence of links and the color represents the value of weight W_{ij} with $i, j = 1, \cdots, 989$.

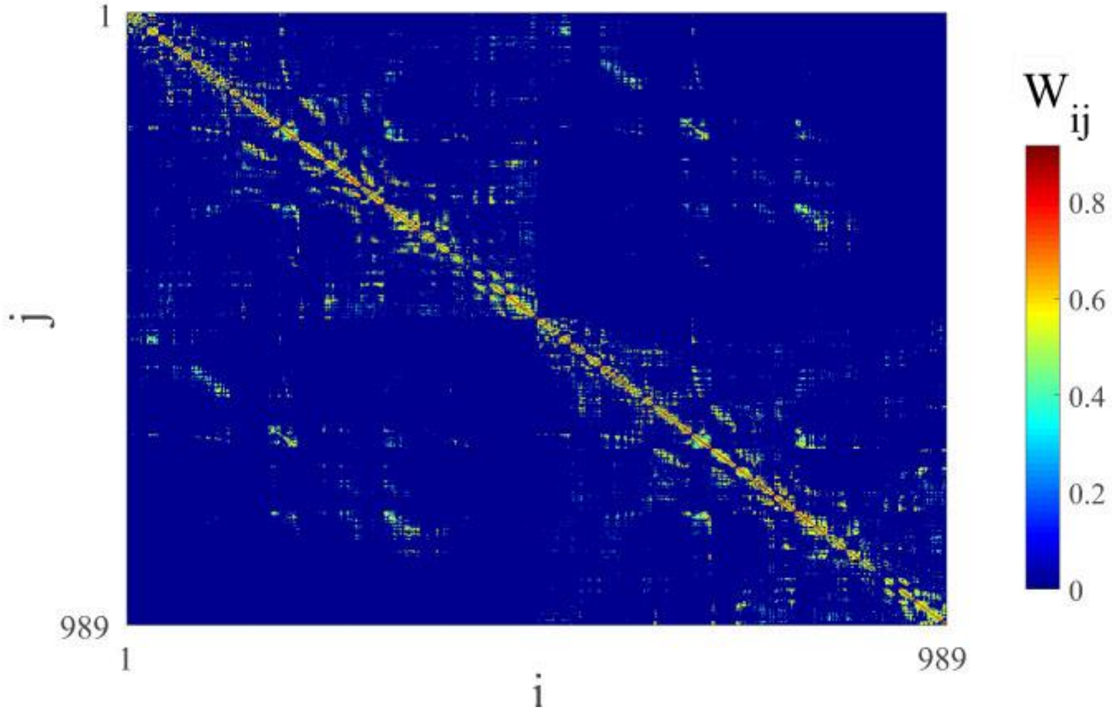

FIG. S1. The weighted connection matrix W_{ij} for the network of cerebral cortex with 989 nodes and 17865 links. Data from Ref. [11, 12] where $i(j)$ represents the $i(j) - th$ ROI with $i, j = 1, \cdots, 989$, the points represent the presence of links and the color represents the weight.

To see the cortical regions of Fig. S1 clear, we put all the 989 nodes on a circle and number them from one cortical area to another one. The 64 regions are equally distributed

on the left and right hemispheres, i.e. 32 on the left hemisphere and 32 on the right hemisphere. We label the nodes in each region consecutively. Then, we put the 17865 links correspondingly into the circle. In this way, the links in the same cortical region, between different regions, and between the left and right hemispheres will not be overlapped, so that it is clear to see how the cortical regions are connected. Fig. S2 shows the topology where the names of anatomical cortical regions are labeled on the circle and the green, blue and red lines represent the links among the nodes within cortical regions, between different regions, and between the left and right hemispheres, respectively.

The brain network can be also represented by the coarse-grained network of 64 cortical regions. For this purpose, we consider two cortical regions I and J be connected if there is at least one link between their nodes. The weight of this inter-regional connection W_{IJ} will be the average weight for all those links between ROIs in the two regions I and J. Fig. S3 shows the coarse-grained network of 64 cortical regions from Fig. S1.

In Table-S1, the columns 1 and 2 show the index and names of the 64 cortical regions, respectively, and the columns 3-5 show the number n_i of nodes in each region-i, the average degree $< k_i >$ of those nodes in the same region-i, and the average intra-degree $< k_i^{in} >$ for those links within the same region- i respectively. The full names for the abbreviated ones in Table-S1 are as follows: each label consists of two parts, a prefix for the cortical hemisphere (r=right hemisphere, l=left hemisphere) and one of 32 designators: BSTS=bank of the superior temporal sulcus, CAC=caudal anterior cingulate cortex, CMF=caudal middle frontal cortex, CUN =cuneus, ENT =entorhinal cortex, FP = frontal pole, FUS = fusiform gyrus, IP = inferior parietal cortex, IT = inferior temporal cortex, ISTC =isthmus of the cingulate cortex, LOCC =lateral occipital cortex, LOF = lateral

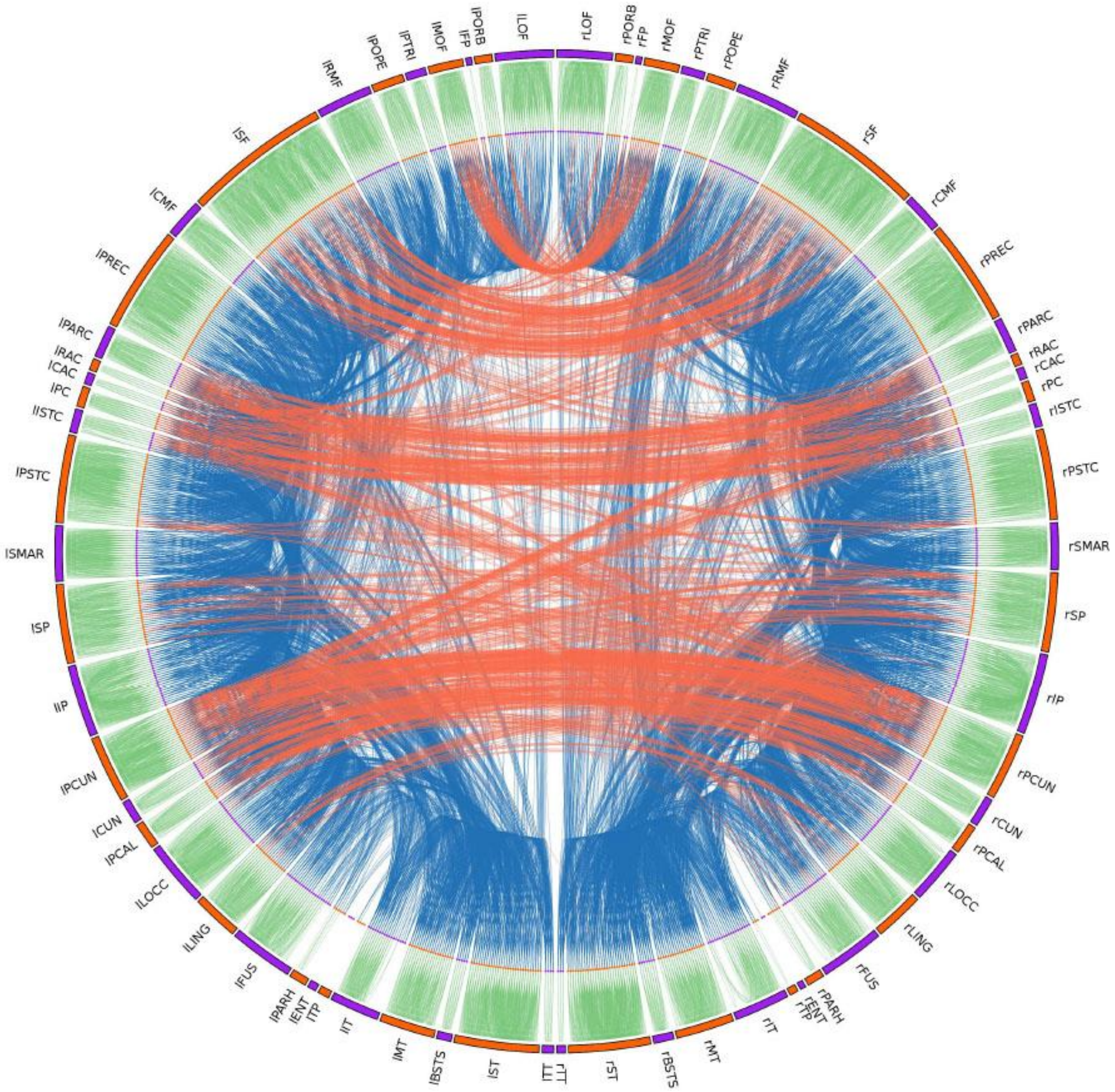

orbitofrontal cortex, LING = lingual gyrus, MOF = medial orbitofrontal cortex, MT = middle temporal cortex, PARC = paracentral lobule, PARH= parahippocampal cortex, POPE= pars opercularis, PORB=pars orbitalis, PTRI=pars triangularis, PCAL=pericalcarine cortex, PSTS = postcentral gyrus, PC = posterior cingulate cortex, PREC = precentral gyrus, PCUN = precuneus, RAC = rostral anterior cingulate cortex, RMF = rostral middle frontal cortex, SF= superior frontal cortex, SP= superior parietal

cortex, ST= superior temporal cortex, SMAR= supramarginal gyrus, TP= temporal pole, and TT = transverse temporal cortex.

FIG. S2. **The network topology of the 64 region parcellation for the network of cerebral cortex with 989 nodes and 17865 links**. The names of functional brain regions are put on the circle and the green, blue and red lines represent the links within cortical region, between different regions, and between the left and right hemispheres, respectively.

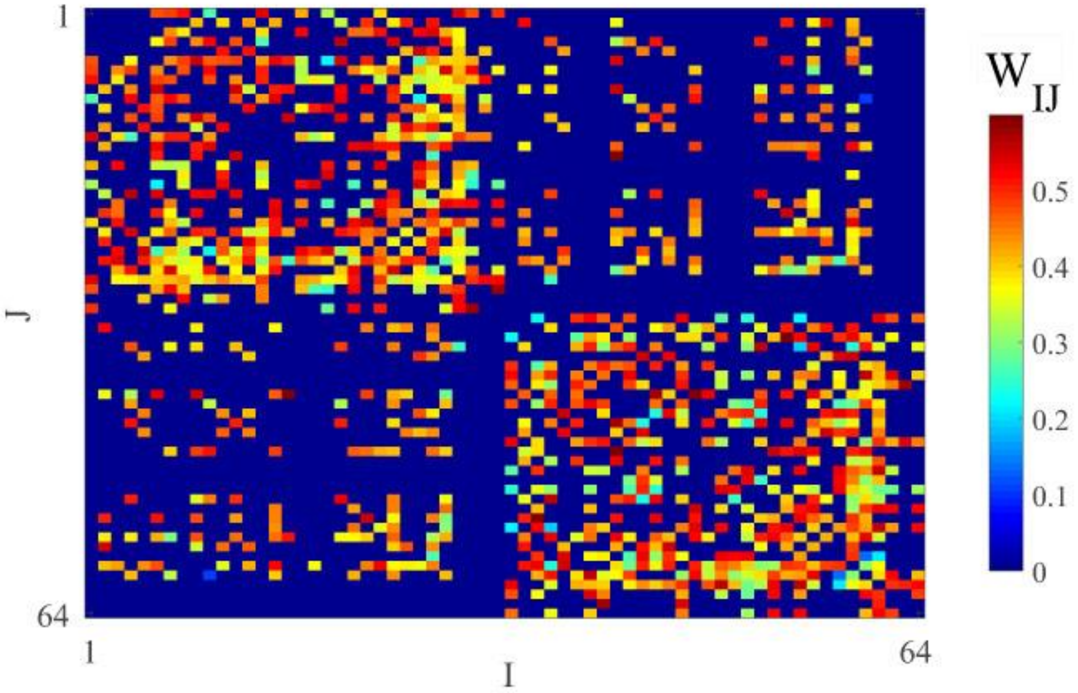

FIG. S3. The weighted connection matrix W_{IJ} for the level of 64 cortical regions. $I(J)$ represents the $I(J)-th$ cortical region with $I,J=1,\cdots,64$, the points represent the presence of links and the color represents the connection weight.

Table S1. Structural parameters of all the 64 cortical regions and their local order parameters for six typical cases. The first and second columns represent the index and their functional names of all the 64 cortical regions, respectively, the third to fifth columns represent the number n_i of oscillators in each region- i, the average degree $<k_i>$ of region- i, and the average intra-degree $<k_i^{in}>$ for those links within the same region-i, respectively, and the columns $6-11$ represent the local R of all the 64 cortical regions for six typical cases with the parameter sets of (c,τ) for cases $1-6$ being $(c=0.075,\tau=15)$, $(c=0.075,\tau=16)$, $(c=0.125,\tau=15)$, $(c=0.125,\tau=16)$, $(c=0.15,\tau=15)$, and $(c=0.15,\tau=16)$, respectively.

Index	ROI	n_i	$< k_i >$	$< k_i^{in} >$	R(case-1)	R(case-2)	R(case-3)	R(case-4)	R(case-5)	R(case-6)
1	rBSTS	7	45.9	5.7	0.665	0.901	0.461	0.935	0.777	0.935
2	rCAC	4	51	3.0	0.812	0.530	0.967	0.636	0.853	0.343
3	rCMF	13	33.4	8.8	0.347	0.207	0.298	0.429	0.365	0.686
4	rCUN	10	50.5	7.2	0.745	0.748	0.795	0.810	0.939	0.964
5	rFP	2	44	1	0.924	0.979	0.852	0.779	0.537	0.484
6	rFUS	21	22.1	7	0.238	0.122	0.553	0.469	0.739	0.751
7	rIP	28	41.8	13.8	0.160	0.288	0.391	0.277	0.171	0.961
8	rIT	19	25.4	5.8	0.565	0.361	0.644	0.490	0.800	0.718
9	rISTC	8	41.8	5	0.479	0.224	0.448	0.435	0.675	0.825
10	rLOCC	19	26.8	10.6	0.627	0.842	0.502	0.838	0.788	0.872
11	rLOF	19	23	11.9	0.677	0.520	0.698	0.826	0.292	0.821
12	rLING	17	29.3	9.6	0.797	0.865	0.863	0.819	0.857	0.940
13	rMOF	12	36.6	10.2	0.866	0.831	0.811	0.662	0.592	0.620
14	rMT	20	42.6	10	0.584	0.403	0.566	0.617	0.431	0.764
15	rPARC	12	46.2	9	0.587	0.733	0.678	0.788	0.514	0.968
16	rPARH	5	8.2	2	0.673	0.646	0.697	0.450	0.0.904	0.892
17	rPOPE	10	31.5	6.4	0.131	0.164	0.372	0.259	0.466	0.646
18	rPORB	6	20.8	3.3	0.438	0.270	0.765	0.325	0.960	0.738
19	rPTRI	8	33.4	6.8	0.555	0.471	0.749	0.650	0.543	0.541
20	rPCAL	10	43.3	7.8	0.830	0.871	0.957	0.882	0.889	0.980
21	rPSTC	31	34.3	12.2	0.396	0.338	0.756	0.472	0.287	0.923
22	rPC	7	55.6	6	0.464	0.685	0.305	0.546	0.586	0.830
23	rPREC	36	34.4	12.4	0.533	0.239	0.458	0.405	0.137	0.779
24	rPCUN	23	59	12	0.222	0.544	0.495	0.561	0.613	0.755
25	rRAC	4	36	3	0.742	0.950	0.321	0.524	0.505	0.717
26	rRMF	22	28.5	11.2	0.447	0.384	0.424	0.567	0.643	0.592
27	rSF	46	36.3	14	0.640	0.624	0.511	0.287	0.523	0.161
28	rSP	27	39.5	11.2	0.397	0.620	0.644	0.591	0.440	0.894
29	rST	28	44.3	16.4	0.637	0.434	0.141	0.451	0.326	0.759
30	rSMAR	16	42.7	11.6	0.592	0.503	0.314	0.697	0.526	0.929
31	rTP	2	10	1	0.501	0.951	0.759	0.994	0.337	0.336
32	rTT	3	34.7	2	0.819	0.978	0.375	0.961	0.600	0.983
33	lBSTS	5	41.4	4	0.980	0.982	0.888	0.955	0.513	0.950
34	lCAC	4	60.8	3	0.545	0.939	0.549	0.219	0.732	0.567
35	lCMF	13	35.1	8.8	0.515	0.478	0.490	0.826	0.702	0.392
36	lCUN	8	55.1	5.5	0.543	0.547	0.448	0.790	0.949	0.859
37	lFP	2	25.5	1	0.781	0.373	0.388	0.707	0.454	0.860
38	lFUS	22	17	7.5	0.484	0.609	0176	0.877	0.647	0.897
39	lIP	25	38	13.3	0.541	0.308	0.441	0.358	0.757	0.663
40	lIT	17	22.2	6.1	0.668	0.685	0.780	0.758	0.494	0.789
41	lISTC	8	53.1	4.8	0.503	0.664	0.503	0.595	0.812	0.623
42	lLOCC	22	29.5	11.2	0.563	0.736	0.236	0.812	0.789	0.871
43	lLOF	20	22.7	11.8	0.818	0.661	0.559	0.792	0.746	0.632
44	lLING	16	33.9	10	0.736	0.727	0.801	0.699	0.800	0.825
45	lMOF	12	36.6	9.8	0.529	0.783	0.605	0.420	0.255	0.517
46	lMT	19	36.1	9.6	0.783	0.820	0.501	0.753	0.649	0.823
47	lPARC	11	42.2	8.4	0.525	0.861	0.505	0.842	0.659	0.967
48	lPARH	6	7.7	1	0.271	0.172	0.881	0.612	0.673	0.721
49	lPOPE	11	28.5	7.5	0.513	0.578	0.552	0.818	0.445	0.253
50	lPORB	6	22.8	4.7	0.225	0.799	0.758	0.339	0.133	0.286
51	lPTRI	7	27.9	5.4	0.861	0.620	0.417	0.837	0.249	0.628
52	lPCAL	9	50.2	6.2	0.606	0.659	0.609	0.562	0.249	0.831
53	lPSTC	30	34.4	12.5	0.390	0.693	0.456	0.634	0.922	0.731
54	lPC	7	63.6	6	0.352	0.788	0.574	0.850	0.724	0.794
55	lPREC	36	33	12	0.293	0.523	0.371	0.565	0.841	0.534
56	lPCUN	23	61.3	14.2	0.336	0.503	0.445	0.763	0.493	0.766
57	lRAC	4	52.8	3	0.690	0.312	0.147	0.349	0.753	0.322
58	lRMF	19	27.4	10.1	0.682	0.515	0.755	0.760	0.386	0.527
59	lSF	50	37.5	13.6	0.591	0.528	0.349	0.347	0.207	0.306
60	lSP	27	39.4	11	0.268	0.389	0.388	0.512	0.430	0.597
61	lST	29	38.9	14.7	0.427	0.646	0.195	0.629	0.810	0.573

Index	ROI	n_i	$< k_i >$	$< k_i^{in} >$	R(case-1)	R(case-2)	R(case-3)	R(case-4)	R(case-5)	R(case-5)
62	lSMAR	19	34.7	13.6	0.773	0.772	0.744	0.707	0.589	0.863
63	lTP	2	3.5	0	0.992	0.918	0.622	0.946	0.420	0.570
64	lTT	4	40.8	3	0.501	0.706	0.773	0.866	0.855	0.428

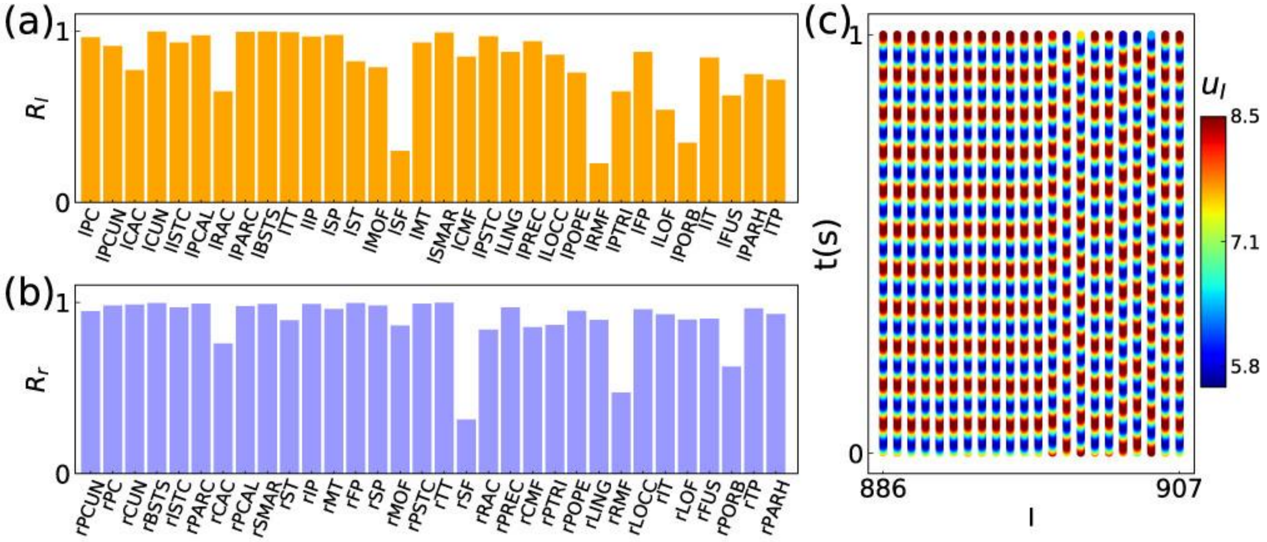

FIG. S4. Local representation of R for the case of Fig. 1(b) in the main text with $c = 0.025$ and $\tau = 16$. (a) and (b) represent R_l and R_r for all the cortical regions in the left and right hemispheres, respectively. (c) The spatiotemporal pattern of a typical region 38 (lFUS) from (a).

B. Local order parameter in individual cortical regions

Fig. 3(a) in the main text shows the local representation of R for the case of $c = 0.075$ and $\tau = 15ms$ and Fig. 3(b) in the main text shows its CS within lFUS. The situation is similar for some other regions in Fig. 3(a) of main text with R clearly smaller than 1.0 and is quite generic for different parameters (c, τ) . We here show one more example corresponding to Fig. 1(b) in the main text, see Fig. S4 where (a) and (b) represent R_l and R_r for all the cortical regions in the left and right hemispheres, respectively. We see that the values of R_l and R_r are different for different cortical regions, suggesting that different regions may show different CS. Furthermore, we take the cortical region 38 (lFUS) with the nodes $886 - 907$ from Fig. S4(a) as an example. Fig. S4(c) shows its spatiotemporal pattern. It can be seen that some of the oscillators are synchronized and the others are incoherent, indicating a CS. Thus, the chimera state in the case of Fig. 1(b) in the main text also shows the feature of *spatial multi-scaled CS*.

A few more examples of local order parameter in individual cortical regions are shown in the Table-S1, where the columns $6 - 11$ represent six typical cases with the parameter sets of (c, τ) for cases $1 - 6$ being $(c = 0.075, \tau = 15)$, $(c = 0.075, \tau = 16)$,

$(c = 0.125, \tau = 15)$, $(c = 0.125, \tau = 16)$, $(c = 0.15, \tau = 15)$, and $(c = 0.15, \tau = 16)$, respectively. It is easy to see that their values of R are also distributed, although they are different from case to case.

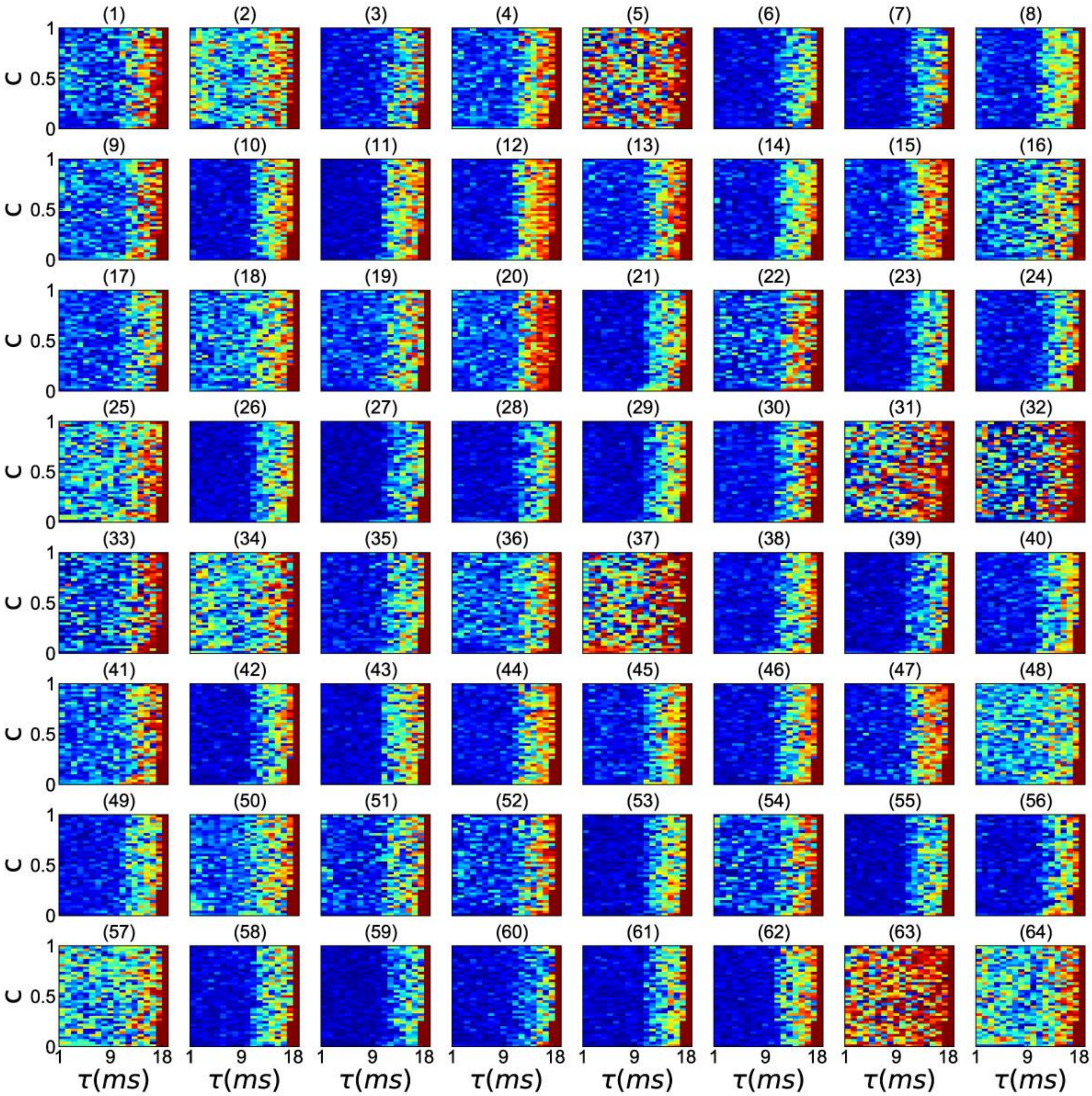

FIG.S5. Phase diagram of R on the parameter space $\tau - c$ plane for all the 64 cortical regions. Each panel represents a specific one of the 64 cortical regions and the numbers are the index of the specific cortical regions.

For reference, we here show all the phase diagram of R of the 64 cortical regions on the parameter space $\tau - c$ plane, see Fig. S5 for the results. We see that the distribution of R can be quite different for different cortical regions, indicating that they take different roles in brain functions and thus guarantee the diversity of CS patterns.

C. Case of distributed time-delays

The time delay τ is approximately considered as a constant in the main text. However, in realistic situation, the time delay τ is not a constant but related to the distance between two nodes. It was pointed out that the axonal conduction delays depend on the distance between neurons in the cerebral cortex and can amount to several tens of milliseconds [3]. Then, an important question is whether the observed results in the main text are robust to the distributed time delay τ . To answer this question, we first need to know how the time delay τ is formed in cerebral cortex.

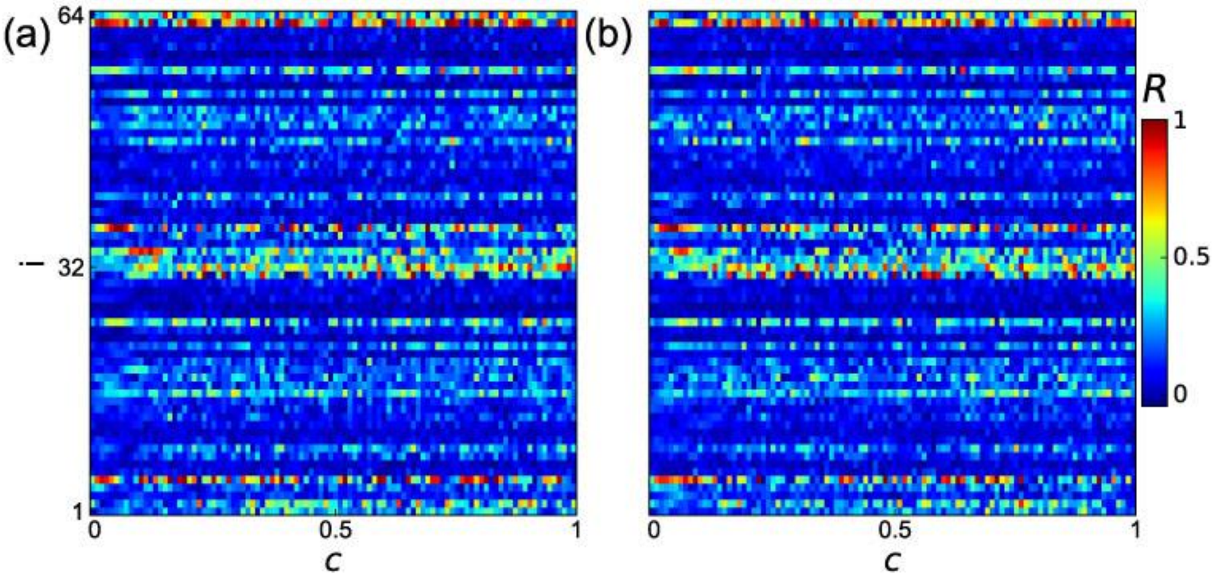

FIG. S6. Local representation of R for the case of distributed τ. i represents the index of local regions and c denotes the coupling strength. (a) and (b) represent the cases of $\tau_{max} = 10ms$ and $20ms$, respectively.

Two key elements of time delay are the speed of signal transmission over links and the distance between two connected nodes, i.e. the length of link. For the first aspect, many studies have been done on both human beings and animals and it is found that the speed of signal transmission is finite. This limited speed of signal transmission is the direct reason to give rise to a finite time delay [3]. For animals, it was pointed out that the axonal conduction velocities are of $1m/s$ for ipsilateral cortico-cortical connections and $1.5m/s$ for callosal cortico-cortical fibers in the rabbit brain [4]. While for the human brain, it was pointed out that signals travel with approximately $1.7m/s$ for myelinated and $0.1m/s$ for unmyelinated fibers. In sum, the conclusion is that the speed of signal transmission is complicated, i.e. depending on agents. For simplicity, we here approximately consider the same speed for all the links in the network of cerebral cortex. For the second aspect, it is difficult to measure the real distance between two connected nodes because of the complicated structure of cerebral cortex such as the gyrus. Especially, the links between the left and right hemispheres are connected by the corpus callosum, which makes the measure more difficult.

Combining these two aspects, we here use an approximate approach, i.e. Euclidean distance, to measure all the link lengths of the network of cerebral cortex and assume that the time delay τ for each link is proportional to its link length. In details, we first calculate a Euclidean distance d_{ij} between connected two nodes i and j. Then, we obtain the time delay τ_{ij} for the link between the two nodes i and j as follows

$$\tau_{ij} = \tau_{max} d_{ij} / d_{max} \quad (1)$$

where d_{max} and τ_{max} represent the maximum of d_{ij} and the maximum of τ_{ij}, respectively. Fig. S6 shows the local order parameter R where the x-axis represents the coupling strength c, the y-axis represents the index of local regions, and (a) and (b) represent the cases of $\tau_{max} = 10ms$ and $20ms$, respectively. It is easy to notice that the two panels of Fig. S6 are similar to each other, indicating that the local order parameter R is robust to the delay parameter τ_{max}.

We also notice from the two panels of Fig. S6 that the preference for R to take larger values is different for different region i. Especially, the regions $5, 31, 32, 34, 37, 63$ and 64 have the largest fraction for R to take larger values. Recall that we have revealed in the main text that the regions $5, 31, 32, 37$ and 63 have the largest values of synchronized n_s. Thus, the regions with the largest fraction of synchronized R in Fig. S6 is highly consistent with the regions with the largest values of synchronized n_s in Fig. 4(a) in the main text, confirming that these specific regions have the most probability to take part in the brain functions.

Further, we would like to check how the distributed τ influences the spatial multi-scaled CS. For this purpose, we arbitrary choose a specific case from Fig. S6(a) with $c = 0.75$ and show all the values of R for the 64 local regions in Fig. S7(a). We see that the regions $2, 32, 37, 48$ and 63 have the largest values of R. Then, we choose a region 45 (lMOF) with a middle value of R to check its dynamical behaviors, shown in Fig. S7(b). We see that it is a coexistence of synchronized cluster and disordered oscillators, indicating that it is a CS. Comparing Fig. S7(b) with Fig. 3(b) in the main text, we see that they are similar each other, confirming the robustness of the spatial multi-scaled CS to the distribution of τ.

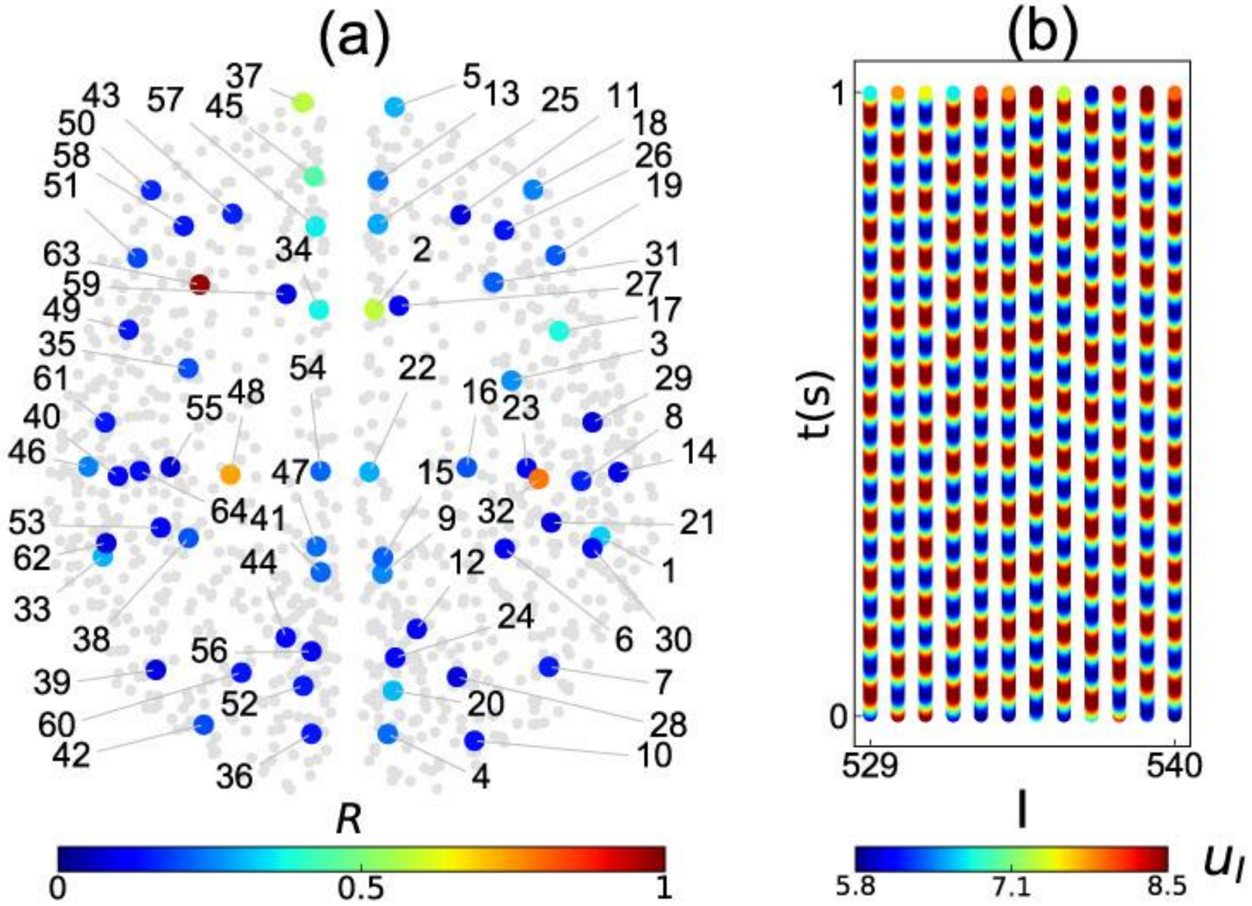

FIG. S7. Spatial multi-scaled CS for the case of distributed τ . (a) Local representation of R for the case of Fig. S6(a) with $c = 0.75$, where the color points with numbers represent the network of the 64 local regions and the gray background points represent the network of 989 nodes. (b) The spatiotemporal pattern of the cortical region 45 (lMOF) from (a).

D. Hierarchy trees of anatomical and functional networks

By the dissimilarity $1 - S_{\ell\ell'}$, Fig. 6(a) and (b) in the main text show the hierarchical trees of both anatomical and functional networks for the case of $c = 0.075$ and $\tau = 15$, where the hierarchy trees of both anatomical and functional networks are divided into four branches. An interesting finding is that the hierarchy tree of anatomical network is closely related to the hierarchy tree of functional network. That is, some dynamical branches match well with the corresponding structural branches, while other dynamical branches are the combination of a few structural branches. Here we examine the relationship at larger scale by dividing the trees into two branches, see Fig. S8. The results show that ST1 contains the whole right hemisphere and part of left hemisphere, while ST2 completely comes from part of the left hemisphere [Fig. S8 (c)]. DT1 is mainly from ST2, but also from part of ST1 [Fig. S8 (e)], while DT2 is mainly from ST1, but also from part of ST2 [Fig. S8 (f)], and DT1 is solely from the left hemisphere [Fig. S8 (d)]. The coherence in the whole right hemisphere and incoherence between different dynamical clusters in the left hemisphere is similar to the situation of unihemispheric sleep in human brain. The results suggest that it is possible under suitable dynamical conditions to have only part of

the hemisphere to be dynamically segregated (here DT1), while leaving the others in a coherent state, including the combination between left and right hemispheres (DT2). The results further confirm that there is a close relationship between the hierarchy trees of anatomical and functional networks that the structural trees provide both constrains on the dynamical clusters (e.g., a structural branch can dominate a dynamical cluster) and the flexibility of combinations (e.g., the structural branches can split and reunite to from different clusters). Our analysis shows that this close relationship between the hierarchy trees of anatomical and functional networks also holds for other cases of chimera states.

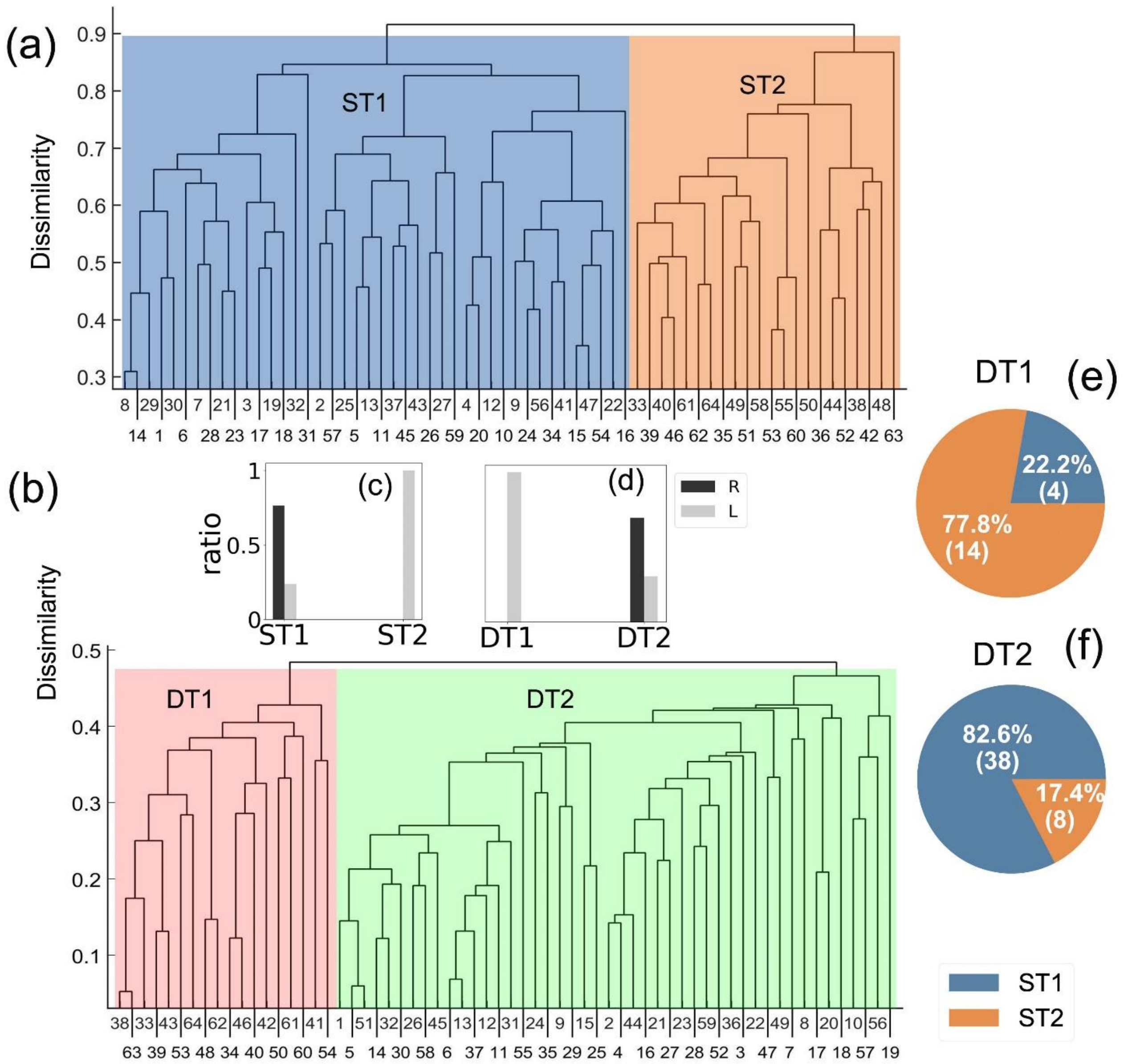

FIG. S8. Relationship between anatomical and functional trees in case of more branches in the level of 64 cortical areas, corresponding to Fig. 6 in the main text with $c = 0.075$ and $\tau = 15$. (a) Hierarchy tree of anatomical network with two branches, i.e. ST1 and ST2. (b) Hierarchy tree of functional network with two branches, i.e. DT1 and DT2. (c) Fractions of nodes from right and left hemispheres in ST1 and ST2 of (a). (d) Fractions of nodes from right and left hemispheres in DT1 and DT2 of (b). (e) and (f)) show the fractions of DT1 and DT2 in (b) coming from ST1 and ST2 of (a), respectively.

REFERENCES

1. P. Hagmann, L. Cammoun, X. Gigandet, R. Meuli, C. J. Honey, J. V. Wedeen, O. Sporns, Mapping the structural core of human cerebral cortex. *PLoS Biol* **6**, e159 (2008).

2. C. J. Honey, O. Sporns, L. Cammoun, X. Gigandet, J. P. Thiran, R. Meuli, P. Hagmann, Predicting human resting-state functional connectivity from structural connectivity. *Proc Natl Acad Sci U S A* **106**, 2035-2040 (2009).

3. M. Dhamala, V. K. Jirsa, M. Ding, Enhancement of neural synchrony by time delay. *Phys Rev Lett* **92**, 074104 (2004).

4. H. A. Swadlow, Efferent neurons and suspected interneurons in motor cortex of the awake rabbit: axonal properties, sensory receptive fields, and subthreshold synaptic inputs. *J Neurophysiol* **71**, 437-453 (1994).
